# Elucidating Mechanisms of Endophytes Used in Plant Protection and Other Bioactivities With Multifunctional Prospects

**DOI:** 10.3389/fbioe.2020.00467

**Published:** 2020-05-15

**Authors:** Ayomide Emmanuel Fadiji, Olubukola Oluranti Babalola

**Affiliations:** Food Security and Safety Niche, Faculty of Natural and Agricultural Sciences, North-West University, Mmabatho, South Africa

**Keywords:** antibiotics, commercialization, drug, metabolites, pathogen

## Abstract

Endophytes are abundant in plants and studies are continuously emanating on their ability to protect plants from pathogens that cause diseases especially in the field of agriculture. The advantage that endophytes have over other biocontrol agents is the ability to colonize plant's internal tissues. Despite this attributes, a deep understanding of the mechanism employed by endophytes in protecting the plant from diseases is still required for both effectiveness and commercialization. Also, there are increasing cases of antibiotics resistance among most causative agents of diseases in human beings, which calls for an alternative drug discovery using natural sources. Endophytes present themselves as a storehouse of many bioactive metabolites such as phenolic acids, alkaloids, quinones, steroids, saponins, tannins, and terpenoids which makes them a promising candidate for anticancer, antimalarial, antituberculosis, antiviral, antidiabetic, anti-inflammatory, antiarthritis, and immunosuppressive properties among many others, even though the primary function of bioactive compounds from endophytes is to make the host plants resistant to both abiotic and biotic stresses. Endophytes still present themselves as a peculiar source of possible drugs. This study elucidates the mechanisms employed by endophytes in protecting the plant from diseases and different bioactivities of importance to humans with a focus on endophytic bacteria and fungi.

## Introduction

Endophytic microorganisms are referred to as the microbes that inhabit the internal parts of a plant. They gain entrance into the seed, leaf, stem, and root of a plant and they are not harmful to the host plant (Yadav, 2018). Endophytes improve plant growth by secreting phytohormones and consequently help in nutrition improvement using bidirectional nutrient transfer and enhancement of the health of plants by protecting them against phytopathogens (Andreozzi et al., 2019; Shen et al., 2019). Plant-endophyte interaction triggers the protection of plants against harmful conditions of the environment such as heavy metal presence and drought (Khan et al., 2019; Kushwaha et al., 2019). Endophytes are numerous and studies have it that they are present in many plants; they became important due to their capacity to produce many bioactive metabolites and biotechnologically relevant enzymes (Khan et al., 2014; Rajamanikyam et al., 2017). Most times when endophytes are inoculated in the plant, they produce significant biomass increment and also help in boosting commercial agriculture (Santoyo et al., 2016; Shen et al., 2019). Endophytes are gaining biotechnological and industrially relevance as a result of their ability to secrete secondary metabolites, serve as biocontrol agents, antimicrobial agents, antitumor agents, and immunosuppressants, and to secrete antiviral compounds and development of natural antioxidants, antidiabetic agents, antibiotics, and insecticidal products (Gouda et al., 2016; Yadav, 2018).

In the last 20 years, endophytes isolated from most plants have shown themselves to be a rich source of natural products for industrial and agricultural use amongst several other applications. Enzymes can be used to replace poisonous chemicals. They thrive best under normal temperatures and neutral pH. As the years' progress, researchers are beginning to see prospects in microbial enzyme production. There are many reports currently that microorganisms isolated from the extreme environments have great biotechnological applications in medicine, agriculture, and industry (Archna et al., 2015; Yadav et al., 2015; Singh et al., 2016; Sahay et al., 2017). This review aimed to present the various mechanisms of action used by endophytes in protecting a plant and report some bioactivities of importance to people with special emphasis on endophytic bacteria and fungi.

## An Overview of Endophytes

The word endophyte connotes “in the plant,” and studies have established that endophytes emanate from the phyllo sphere and rhizosphere (Verma et al., 2017). Endophytes are generally isolated from the internal tissues of plants after surface sterilization. Plant association with microorganisms may be classified in many forms such as mycorrhiza, pathogenic, epiphytic, saprotrophic, and endophytic based on the type of colonization and their roles (Brader et al., 2017). Only a few microorganisms such as endophytic microbes and mycorrhiza fungi can be exceptional and find their way into the inner tissues of a plant. Endophytic microorganisms such as bacteria, fungi, eukaryote, and archaea inhabit plant tissues (de Tender, 2017), they are known not to cause any harm to the host plant. They exhibit a symbiotic association with tissues of most plants and sometimes can be slightly pathogenic. These endophytic microbes have been identified in many varieties of plants some of which are Rice, Wheat, Tomato, Cowpea, Maize, Strawberry, Chickpea, Mustard, Sugarcane, Chili, Citrus, Soybean, Cotton, Pearl millet, and Sunflower (Verma et al., 2017; Yadav et al., 2018).

The advent of microbial biotechnology has helped in establishing the fact that microorganisms play significant roles in industry, agriculture, and medicine (Gouda et al., 2016; Rajamanikyam et al., 2017). Having a better understanding of the diverse roles microorganisms play in the ecosystem will enhance the ways they can be applied in the field of agriculture most importantly for plant growth and crop yield (Nair and Padmavathy, 2014). The world of endophytes has attracted the interest of many researchers due to their significant roles in promoting growth and in enhancing the survival of plants under extreme conditions (Shen et al., 2019). Bioactive metabolites secreted by endophytic microorganisms are useful in industries, agriculture, and the field of medicine. Plants perform a major function of determining the type of microorganism that can be associated with it by the makeup of its root exudates (Andreozzi et al., 2019). Thus, the interaction between endophytic microorganisms and plants greatly depends on the capacity of these microbes to use the exudates produced by the plant roots as their energy source (Kandel et al., 2017). Endophytes can efficiently enhance growth promotion using different modes of operations and increasing the resistance of plants to extreme conditions (Yadav, 2018). Notably, endophytic microbes have been used in the mass production of industrially relevant products such as antibiotics, enzymes, and riboflavin among others (Latz et al., 2018). The resistance to antibiotics is on the increase especially among organisms that cause disease and this has great public health implications if proper care is not taken (Adegboye et al., 2012).

Microbial biotechnology has gone beyond the production of only metabolites such as ethanol and butanol, now biotransformation of many chemicals has been incorporated to reduce the impact of environmental pollution using different strategies such as bioremediation, waste management, and composting. For some decades now, attention has been shifting to the use of microorganisms, animals, and plants for the production of new drugs (Gouda et al., 2016; Latz et al., 2018). These products, mainly from natural sources, are less toxic and cheap. Endophytic fungi have a great prospect for the secretion of numerous bioactive metabolites. Some of these phytonutrients or metabolites like polyphenol and anthocyanin can reduce diseases such as cancer and heart diseases.

## General Mechanisms Employed by Endophytic Bacteria and Fungi in Plant Protection

**Figure 1:**
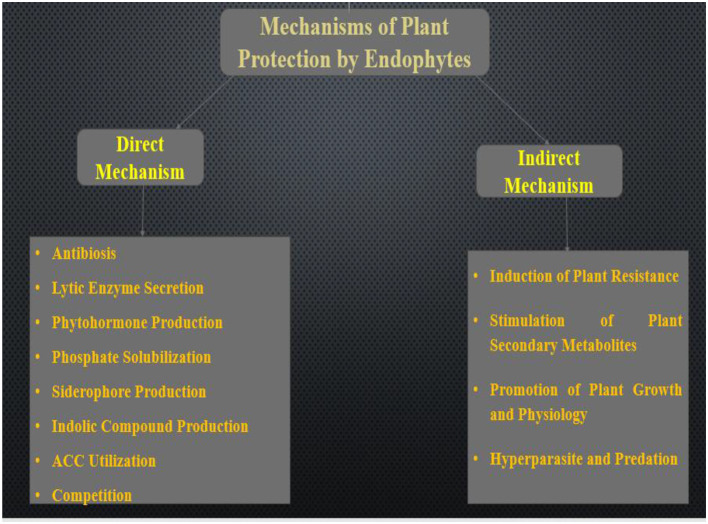
Mechanisms employed by endophytes for plant protection.

Endophytic microorganisms help in boosting plant fitness through several mechanisms of action. The generally mechanisms employed by endophytic bacteria and fungi was discussed in this section. The modes of action include direct and indirect mechanisms as illustrated in Figure 1. These mechanisms were discussed in detail below.

## Direct Mechanisms of Plant Protection From Pathogens

Recent studies carried out on endophytes have established their capacity to enhance host defense against diseases and reduce the damages attributed to pathogenic microorganism (Ganley et al., 2008; Mejía et al., 2008). The most common strategy employed by these researchers is *in vitro* direct plate antagonistic reaction against pathogens or by comparing the rate of survival of plant inoculated with control. Although some studies have presented new mechanisms used by endophyte in reducing the effects of pathogens, current knowledge about endophytes, pathogen, and plant regulations still not fully understood (Ganley et al., 2008). In this section, we shall be discussing a direct mechanism (endophytes-pathogens interactions) and indirect mechanism (enhanced plant defense). In the direct mechanism, endophytes directly produce antibiotics which help in suppressing pathogens. However, direct endophyte-pathogen interactions are compounded and responsive to species-specific antagonism (Arnold et al., 2003). Some examples of direct mechanisms used by endophytes are discussed below.

## Antibiotics Produced by Endophytes

Most endophytes have been reported to produce some secondary metabolites and some of them exhibit antibacterial and antifungal properties which help in inhibiting the growth of phytopathogenic microorganisms (Gunatilaka, 2006). Many types of research are still ongoing in a bid to identify endophyte metabolites for possible commercial use. Different bioactive compounds have been studied for their ability to inhibit many phytopathogens (Suryanarayanan, 2013; Daguerre et al., 2016). Also, many metabolites with antimicrobial properties have been discovered from endophytes, some recently reviewed one are flavonoids, peptides, quinones, alkaloids, phenols, steroids, terpenoids, and polyketides (Mousa and Raizada, 2013; Lugtenberg et al., 2016). When many microbial species are present in the same plant, the association propels the secretion of metabolites by the endophytes or the host to inhibit the growth of microbes that are harmful (Kusari et al., 2012). In some instances, the endophytes and the host plant do use some distinct pathways in enhancing the production of metabolites, some use induced metabolism which helps in metabolizing the product of the other (Kusari et al., 2012; Ludwig-Müller, 2015). It was later concluded that many endophytic strains cannot produce the compounds independently (Heinig et al., 2013).

An endophyte isolated from *Cassia spectabilis*, named *Phomopis cassia* was able to synthesize five substances similar to 3,11,12-trihydroxycadalene and cadinane sesquiterpenes in which one of the five derivatives produced the most active antifungal metabolite against *Cladosporium cladsporioides* and *Cladosporium sphaerospermum* (Silva et al., 2006). Alkaloids were reported to have strong potential in inhibiting the proliferation of microbes, for instance, altersetin, a novel alkaloid which was isolated from the endophyte *Alternaria* spp., exhibited a strong antibacterial effect against many gram-positive bacteria that are pathogenic (Hellwig et al., 2002). Another metabolite which exhibited antibiosis is a volatile oil. An endophytic fungus from the tropical trees known as *Muscodor albus* produced many volatile organic compounds, including aciphyllene, 2-butanone and 2-methyl furan which were reported to produce antibiotic properties (Atmosukarto et al., 2005). Also, fungal endophytes isolated *in vitro* from *Artemisia annua* can suppress the growth of most phytopathogenic organisms by the production of antifungal compounds such as n-butanol and ethylacetate (Liu et al., 2001). Tian et al. (2017) assessed the role of anti-fungal protein produced by *Epichloë festucae* in controlling *Sclerotinia homoeocarpa* in *Festuca rubra*. The result presented this attribute by fescues as one of the unique ones. The mechanism of plant protection used by Paraconiothyrium strain SSM001 linked with the production of taxol from yew tree (*Taxus* spp.) against dangerous wood-decaying fungi was investigated by Rafiqi et al. (2013) and Soliman et al. (2015). A summary of related studies on the antimicrobial properties of endophytes is presented in Table 1.

**Table 1:** Summary of studies on the antimicrobial activities of endophytes.

**Endophytes**	**Plant host**	**Activity**	**Compounds**	**Class of compound**	**References**
**Endophytic bacteria**					
*B. subtilis*	–	Antifungal	Bacilysocin	Phospholipid	Tamehiro et al., 2002
*B. substilis*	*Allanmands cathartica*	Antifungal	Terpene	Terpenoids	Nithya and Muthumary, 2011
*B. substilis*	–	Antibacterial	Subtilin	Peptides	Singh et al., 2017
*Bacillus atrophaeus, Bacillus mojavensis*	*Glycyrrhiza uralensis* (Licorice)	Antifungal	1,2-bezenedicarboxyl acid, Methyl ester, Decanodioic acid, bis(2-ehtylhexyl) ester	Polyketides	Mohamad et al., 2018
*Lysinibacillus, Staphylococcus, Enterobacter, Pseudomonas*, and *Bacillus species*	*Combretum molle*	Antibacterial	–		Diale et al., 2018
*B. licheniformis, B. subtilis* subsp*. Inaquosorum*, and *B. pumilus*	*Moringa peregrina*	Antibacterial and antifungal	–		Aljuraifani et al., 2019
**Endophytic fungi**					
*Phoma* sp.	*Cinnamomum mollissimum*	Antifungal	5-hydroxyramulsin	Polyketides	Santiago et al., 2012
*Geotrichum candidum, Cylindrocladium* sp*. Fusarium* sp*. Cladosporium cladosporioides* sp*., Mucor pusillus, Rhizopus* sp., and *Alternaria alternata*	*Phyllanthus reticulatus* Poir	Antibacterial and antifungal	–		Pai and Chandra, 2018
*Phompsis* sp.	*Aconitum carmichaeli*	Antifungal	Gavodermside and Clavasterols	Steroids	Wu et al., 2013
*Xylaria* sp. F0010	*Abies holophylla*	Antifungal	Griseofulvin	–	Park et al., 2005
*Chaetomium globosum*	*Ginkgo biloba*	Antifungal	Chaetomugilin A and D	Azaphilone derivative	Qin et al., 2009
*Pestalotiopsis mangiferae*	*Mangifera indica* Linn	Antibacterial	4-(2,4,7-trioxa-bicyclo[4,10]-heptan-3-yl)	Phenols	Subban et al., 2013
*Aspergillus* sp.	*Bauhinia guianensis*	Antibacterial	Fumigaclavine C and Pseurtotin C	Alkaloids	Pinheiro et al., 2013
*Phomopsis* sp*., Botryosphaeria* sp.	*Garcinia* sp.	Antibacterial and antifungal	–	–	Phongpaichit et al., 2006
*Nigrospora sphaerica (URM-6060)* and *Pestalotiopsis maculans (URM-6061)*	*Indigofera suffruticosa Miller*	Antibacterial	–	–	Santos et al., 2015
MR1B and MRB.2	*Catharanthus roseus* and *Euphorbia hirta*	Antibacterial and antifungal	Citreoisocoumarin, paxilline, nigricinol, fatty acid, sceptrin, cladosporin	Isocoumarin derivative	Akpotu et al., 2017
**Endophytic actinomycetes**					
*Streptomyces noursei*	–	Antifungal	Nystatin	Steroids	Fjærvik and Zotchev, 2005
*Streptomyces* sp.	–	Antibacterial	Harmaomycin	Peptide derivatives	Bae et al., 2015
*Streptomyces remosus*	–	Antifungal	Tetracyclin	Steroids	Nelson, 2001
*Streptomyces* sp.	*Grevillea pteridifolia*	Antibacterial	Kakadumycin A Echinodermycin	Peptides	Castillo et al., 2003
*Streptomyces* sp. TP-A0595	*Allium tuberosum*	Antifungal	6-Prenylindole	Alkaloids	Singh and Dubey, 2018
*Aeromicrobium ponti*	*Vochysia divergens*	Antibacterial	1-Acetyl-β-carboline, Indole-3-carbaldehyde, 3-(Hydroxyacetyl)-Indole, Brevianamide F, and Cyclo-(L-Pro-L-Phe)	Alkaloids	Gos et al., 2017
*Streptomyces* sp. neau-D50	*Glycine max*	Antifungal	3-Acetonylidene-7-Prenylindolin-2-one and 7-Isoprenylindole-3-carboxylic acid	Alkaloids	Zhang et al., 2014
*Actinosynnema pretiosum*	*Maytenus serrata*	Antibacterial	Ansamitocin	Polyketides	Siyu-Mao, 2013
*Streptomyces* sp. TP-A0456	*Aucuba japonica*	Antibacterial	Cedarmycin A and B	Terpenes and Terpenoids	Sasaki et al., 2001
*Streptomyces aureofaciens* CMUAc130	*Zingiber officinale*	Antifungal	5,7-Dimethoxy-4-pmethoxylphenylcoumarin; 5,7-Dimethoxy-4-phenylcoumarin	Coumarins	Taechowisan et al., 2007
*Streptomyces* sp. BT01	*Boesenbergia rotunda* (L.)	Antibacterial	7-Methoxy-3, 3′,4′,6-tetrahydroxyflavone and 2′,7-Dihydroxy-4′,5′-Dimethoxyisoflavone, Fisetin, Naringenin, 3′-Hydroxydaidzein, Xenognosin	Flavonoids	Taechowisan et al., 2014
*Streptomyces* sp. DSM 1175	*Alnus glutinosa*	Antibacterial	Alnumycin	Quinones	Singh and Dubey, 2018
*Dactylosporangium* sp. strain SANK 61299	*Cucubalus* sp.	Antifungal	Streptol	Tannins	Singh and Dubey, 2018
*Verrucosispora maris* AB-18-032	*Sonchus oleraceus*	Antibacterial	Proximicin	Peptides	Fiedler et al., 2008

## Lytic Enzymes Secretion

Most microorganisms secrete lytic enzymes for the hydrolysis of polymers (Gao et al., 2010). About 1,350 compounds can be secreted; among them are cellulose, hemicellulose, proteins, DNA, and chitin (Tripathi et al., 2008). For endophytes to colonize the surface of plants, they produce numerous enzymes which successively aid the hydrolysis of the plant cell wall. These enzymes help in reducing phytopathogens indirectly and also aid the fungi cell wall degradation. There are numerous types of enzymes some of which are chitinases, cellulases, hemicellulases, and 1, 3-glucanases. Application of mutagenesis to the genes of 1, 3-glucanase present in a strain of *Lysobacter enzymogenes* reduced the biocontrol activity toward the damping-off disease of sugar beet caused by Pythium and tall fescue leafspot disease (Gao et al., 2010). The lytic enzymes produced by Streptomyces have a strong effect on antagonizing cacao witches broom disease (Macagnan et al., 2008). Even though enzymes may not be solely effective as an antagonizing agent, they may enhance antagonistic activities when combined with other mechanisms. Pectinase was also reported to aid the reduction of pathogenesis in a plant (Babalola, 2007).

## Production of Phytohormone

Endophytes produce phytohormone which enhances plant growth promotion and changes the morphology and structure of the plant. As a result of this attribute, endophytes have gained ground in the area of agricultural sustainability (Sturz et al., 2000). The mechanism adopted by endophytes in the production of phytohormones in the host plant is related to the mechanism used by rhizobacteria in plant growth promotion. They help in growth promotion and protection of non-leguminous plants by the secretion of gibberellic acid (Khan et al., 2014), auxins (Dutta et al., 2014), indole acetic acid (Khan et al., 2014; Patel and Patel, 2014), and ethylene (Babalola, 2010; Kang et al., 2012).

Indole acetic acid (IAA) triggers plant cell division, differentiation and extension; stimulates of seed and tuber germination; increases the rate at which root and xylem develop, enhances lateral initiation, controls the rate of vegetative growth, and the formation of adventitious root formation; aw well as the formation of pigments and biosynthesis of metabolites, controls responses to gravity, light, and fluorescence, affects photosynthesis and resistance to extreme conditions (Gao et al., 2010). IAA secreted by plant growth-promoting bacteria sometimes slows down the physiological processes listed above by affecting the level of auxin secretion by the plant. Also, the IAA produced by endophytic bacteria has the capacity to increase the root length and surface area, thereby giving room for the plant to have better access to nutrients from the soil. Additionally, IAA production expands bacteria cell walls and increases the secretion of exudates alongside providing more nutrients for growth enhancement of other beneficial bacteria present in the rhizosphere. Therefore, the IAA produced by endophytic bacteria is recognized as the major effector molecule in phytostimulation, pathogenesis, and plant-microbe interaction (Gao and Tao, 2012). Several have studies demonstrated that endophytic actinomycetes also produce plant growth-promoting compounds such as IAA which have been reported to enhance the formation and elongation of plant adventitious roots in a plant (de Oliveira et al., 2010; Shimizu, 2011).

## Phosphate Solubilization

The third most important nutrient for plant growth is potassium (K) and endophytes are capable of solubilizing forms of potassium that are insoluble. Most soil-related microorganisms are capable of solubilizing insoluble phosphate to enhance the production of P, thus making it available for plant use (Alori et al., 2017). The most common mechanism used for inorganic phosphate solubilization is the dissolution of mineral compounds such as organic acids, protons, siderophores, carbon dioxide (CO_2_), and hydroxyl ions (Olanrewaju et al., 2017). The existence of microorganisms that solubilizes potassium might have opened our eyes to an alternative means of making potassium available for plant uptake (Rogers et al., 1998). Endophytes also introduce organic acids into the soil which help to solubilize the phosphate complexes and change them into ortho-phosphates for plant absorption and usage. Numerous bacteria species namely *Bacillus mucilaginosus, B. circulans, Pseudomonas* sp., *Burkholderia, Paenibacillus* sp., *Acidothiobacillus ferrooxidans*, and *Bacillus edaphicus* were identified in the release of the accessible form of potassium from potassium-bearing minerals in soils (Yadav, 2018). As abundant as phosphorus is in the soil, unfortunately, many of its remains do not exist in an insoluble form (Miller et al., 2010). Many studies have shown the role of endophytic microorganisms as a biofertilizer and biocontrol agent. For example, endophytes isolated from the root nodule for peanut, identified as *Pantoea* spp. was reported to have strong solubilizing activity (Yadav et al., 2018). Similarly, endophytic actinomycetes have been reported to perform an important role in phosphate solubilization and also enhances its availability to plants through chelation, acidification, and mineralization and redox changes of organic phosphorus (Singh and Dubey, 2018). Solubilization of phosphate alongside secretion of phytase was demonstrated by an endophytic actinomycete, *Streptomyces* sp., which significantly improve plant growth (Jog et al., 2014).

## Siderophore Production

Siderophores are small molecular compounds which are capable of chelating iron which can be produced by endophytes and can make iron available for plant use while starving pathogens of iron (Yadav, 2018). Some of the siderophores known to be produced by endophytes can confer biocontrol activities such as hydroxymate, phenolate and/or catecholate types (Rajkumar et al., 2010). Also, the iron-deficient plant is enhanced by siderophores which help in the fixing of nitrogen since diazotrophic organisms require Fe^2+^ and Mo factors for the functioning and synthesis of nitrogenase (Kraepiel et al., 2009). There are many literature evidences to support the insecticidal properties of endophytes (Azevedo et al., 2000). Some endophytes reduce pest penetration of the stele by thickening the endodermal cell wall (Gao et al., 2010). Others destroy insects by producing secondary metabolites. Though some toxic metabolites are traceable to endophytes some of these metabolites are pyrrolizidine, alkaloids, pyrrolopyrazine alkaloid, peramine ergot alkaloid, and ergovaline (Wilkinson et al., 2000).

In the case of plant growth-promoting bacteria, Fe^2+^ is oxidized to Fe^3+^-siderophore complex in the bacterial membrane, which is later introduced into the cell by endophytes through a gating mechanism (Gao et al., 2010). The concentration of soluble metals increases when siderophores bind to the metal surface (Rajkumar et al., 2010). Once the level of heavy metal contaminants is removed, different mechanisms are employed by plants to ingest iron from bacterial siderophores, for example, iron chelates aid the direct absorption of siderophore-Fe complexes, or ligand exchange (Schmidt, 1999). A siderophore-producing endophyte, *Pseudomonas* strain GRP3 was tested on *Vigna radiate* for iron nutrition and the result showed that after 45 days, the plants showed a reduction in iron and chlorotic symptoms, while there was an increase in the content of chlorophyll a and chlorophyll b when the plant was inoculated with strain GRP3 as compared to the control (Sharma et al., 2003). Some endophytic actinomyces such as *Streptomyces* sp. GMKU 3100, *Streptomyces* sp. mhcr0816, *Streptomyces* sp. UKCW/B, and *Nocardia* sp. have been reported to produce siderophores (Singh and Dubey, 2018). Similarly, *S*. *acidiscabies E13* was also reported as a superb producer of siderophore which enhances the growth of Vigna unguiculata under nickel stress conditions (Sessitsch et al., 2013).

## 1-Aminocyclopropane-1-Carboxylate (ACC) Utilization

Generally, ethylene is an essential metabolite for the normal growth and development of plants (Khalid et al., 2006). This important hormone known for enhancing plant growth is secreted by almost all plants and is affected by different abiotic and biotic activities in the soil which improve physiological changes in most plants. The occurrence of extreme conditions such as pathogenicity, drought, salinity, and heavy metals increases the level of ethylene which has side effects on the growth of the plant; this may result in alteration of the cellular processes and defoliation which affects the yield of the crop (Bhattacharyya and Jha, 2012). Many endophytic bacterial species that can produce ACC deaminase have been discovered in genera like *Achromobacter, Agrobacterium, Acinetobacter, Bacillus, Enterobacter, Pseudomonas, Serratia, Ralstonia, Rhizobium, Alcaligenes, Burkholderia* etc. (Kang et al., 2012). Most of the bacterial endophytes trap the ethylene precursor of ACC and change it into ammonia and 2-oxobutanoate (Arshad et al., 2007). Lugtenberg and Kamilova (2009) reported that some stresses like radiation, heavy metals, flooding resistance due to stress coming from polyaromatic hydrocarbons, high light intensity, wounds, high salt concentration, insect predation, draft, and extreme temperature can be overcome by plants that can produce ACC deaminase.

## Competition With Pathogens

Competition is a strong mechanism used by endophytes in preventing pathogens from colonizing the host tissue (Martinuz et al., 2012). Endophytes possess the ability to colonize many plant tissues systemically or locally (Latz et al., 2018). For example, they act through colonization and the lurking of nutrients that are available and by occupying the position that is available for pathogens to carry out their activities (Rodriguez et al., 2009). This can be further buttressed using a study by Mohandoss and Suryanarayanan (2009), who discovered that destruction of endophytes in mango leaves by the application of fungicides in its treatment allows other fungi to inhabit the niche, especially pathogenic fungi.

The mechanism used for competition by most endophytes usually takes place in combination with other mechanisms, instead of acting independently. Since the control method employed by endophytes is often local, they will, however, need to systematically colonize the part of the host where most pathogens may attack. The colonization of the root of oilseed rape with endophyte *Heteroconium chaetospira* could not successfully prevent clubroot symptoms (Lahlali et al., 2014). The result, therefore, indicates the limitations that may be encountered with competition as a biocontrol method, as it may be inactive when there is a high presence of microorganisms causing disease. The symptoms of *Phytophthora* sp. were successfully reduced when treated through a foliar application with mixtures of endophytes from leaves of cacao tree leaves, thus showing competition as one mechanism of disease suppression in a plant. However, some of the strains were also observed to produce other active metabolites which is an indication that, competition might not be the only mechanism used in controlling the disease (Arnold et al., 2003).

## Indirect Mechanisms of Plant Protection From Pathogens

Plants employ several mechanisms to survive in extreme conditions such as drought, salt stress, and cold. Some of the rapid noticeable biochemical and morphological changes observed include the hypersensitive response, cellular necrosis and phytoalexin production. In long term evolution, non-specific (general) resistance and specific resistance are examples of innate resistance developed for pathogen resistance (Kiraly et al., 2007). Those that possess specific resistance can resist infection from one or a few pathogens while the non-specific resistance is active against many pathogens. Endophytes increase the plant defense mechanism through the production of secondary metabolites and enhanced resistance.

## Induction of Plant Resistance

For over 20 years now, many studies have concentrated on the way plants respond to attack from parasites and pathogens using various categories. Induced systemic resistance (ISR) and Systemic acquired resistance (SAR) are the two resistance patterns which have attracted the most attention of researchers. ISR, which is induced by some non-pathogenic rhizobacteria, is moderated by ethylene or jasmonic acid which cannot be linked with the building up of pathogenesis-related (PR) proteins. SAR, which is caused by infections from pathogens is mediated by salicylic acid and linked with the building up of PR proteins (Tripathi et al., 2008). These PR proteins have many enzymes, such as 1, 3-glucanases and chitinases which help in the direct lysing of invading cells, and strengthening of cell wall boundaries to build resistance against infection and cell death (Gao et al., 2010). ISR produced by endophytes can also be linked with the enhancement of genes that are expressed in pathogenesis. The root of tomato harbors important endophytes called *Fusarium solani* which prompt ISR against *Septoria lycopersici*, the causative agent of tomato foliar pathogens and activate PR genes, PR7, and PR5 activities in the roots (Kavroulakis et al., 2007). Redman et al. (1999) reported that the inoculation of a non-pathogenic mutant strain of *Colletotrichum magna* on *Cucumis sativus* and *Citrullus lanatus* produced a high amount of peroxidase, lignin deposition, and phenylalanine ammonialyase which help in protecting the plant against diseases which are caused by *Fusarium oxysporum* and *Colletotrichum orbiculare*. Reduction in the lesions on leaves was observed when *Neotyphodium lolii* engaged against four different pathogens, which could be attributed to enhanced peroxidase and superoxide dismutase activities of the host plant (Tian et al., 2008).

## Plant Secondary Metabolites Stimulation

Secondary metabolites from plants are compounds which have limited functions in the life cycle of the plant but are of great importance in its adaption to different environments (Bourgaud et al., 2001). Notable among all the secondary compounds produced by a plant is an antimicrobial molecule with a low molecular weight called phytoalexins (Gao et al., 2010). It has many substances in it, some of which are terpenoids and flavonoids among many others. *Orchis morio* and *Loroglossum hircinum* were the first to produce phytoalexins in response to a fungal attack initiated by a French botanist called Noel Bernard, outcomes of other studies showed that phytoalexins can now be produced through some abiotic stress factors such as heavy metals ion, salt stress and UV light (Gao et al., 2010). Some studies have concentrated on the production of phytoalexins when triggered by pathogens (Pedras et al., 2008). The production of plant secondary metabolism moderated by endophytes is still a new research area. Findings revealed that the elicitors of *Fusarium* E5 could propel triterpene and dipertene production in cell suspensions of *E. pekinensis*. Li and Tao (2009) reported a similar result in *Taxus cuspidate* culture suspensions, in which culture supernatants of endophytes resulted in increased production of paclitaxel when compared with the control. It is suspected that the co-culturing with elicitor endophytes is a likely way of increasing plant secondary metabolites and boosting plant resistance. Endophytic colonization induced the production of hydrolase for plant cells to reduce the growth of fungi, therefore making endophytes act as elicitors through hydroxylation production (Gao et al., 2010). Some elicitors like glycoprotein, polysaccharides and lipopolysaccharides trigger plant defense mechanisms and increase secretion of plant secondary metabolites which effectively reduce attack by pathogens. However, there is limited information as regards the way in which endophytes survive in the host plant when producing large quantities of secondary metabolites are produced (Gao et al., 2010).

## Promotion of Plant Growth and Physiology

Endophytes sometimes support the host plant defense mechanism against plant pathogenic microorganisms by taking over the plant physiology (Gimenez et al., 2007). As the growth of the plant increases, it develops vigor and resistance to different stresses both abiotic and biotic, this is considered as one of the strategies used by the plant for defense against pathogens (Kuldau and Bacon, 2008). Several studies have shown that plants inoculated with endophytes recorded an increase in growth, drought resistance (Gao et al., 2010), and tolerance to any type of soil (Malinowski et al., 2004). Plant growth can be enhanced by several compounds, an endophyte, *Colletotrichum* sp., isolated from *A. annua* produces a substance called indole acetic acid (IAA) which helps in regulating plant physiology (Lu et al., 2000). Dai et al. (2008) reported that extracts from *Fusarium* sp. E5 produced auxin. Another mechanism adopted by endophytes can be said to be the release of phytohormones (Dai et al., 2008). We can, therefore, believe that plant growth promotion when triggered by endophytes will indirectly protect the plant against pathogens.

## Hyperparasites and Predation

Hyperparasites is another mechanism endophyte use to protect their host ecologically. In this mechanism, endophytes directly attack identified pathogens or their propagules (Tripathi et al., 2008). Endophytic fungi capture the pathogens by twisting and penetrating their hyphae and by the production of lyase which destroys the cell wall of the pathogen. For instance, *Trichoderma* sp. was able to capture and penetrate the hyphae of *Rhizoctonia solani*, a known plant pathogen; the observation was linked to biocontrol activities (Grosch et al., 2006). Another mechanism is microbial predation; this entails a general way of reducing pathogens of plants. Most endophytes exhibit their predatory characteristics in nutrient-deficient conditions. As an example, a variety of enzymes attacking the cell wall of fungal pathogens directly are produced by *Trichoderma* sp. (Gao et al., 2010).

## Endophytic Bacteria and Fungi as Producers of Bioactive Compounds and Bioactivities of Importance to Man

**Figure 2:**
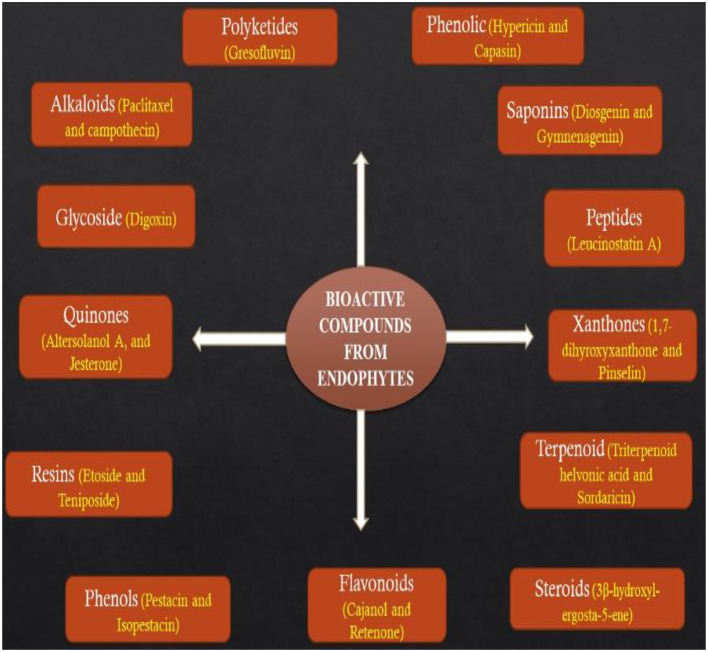
Bioactive compounds produced by endophytes.

Several reports have noted that bioactive metabolites secreted by endophytes are great sources of drugs for the treatment of different types of ailments and their potential applications in food, agriculture, medicine, and cosmetic industries cannot be underestimated (Godstime et al., 2014; Shukla et al., 2014). The metabolites secreted by endophytes are classified into different functional groups such as alkaloids, terpenoids, flavonoids, benzopyranones, tannins, phenolic acids, quinones, steroids, tetralones, and chinones (Figure 2) (Joseph and Priya, 2011; Godstime et al., 2014). Many factors have been reported to affect the way metabolites are extracted from endophytes, some of them are the climatic condition, the season of sample collection and geographical location (Shukla et al., 2014). With the recent developments in the synthetic process, extraction of metabolites from a natural source is becoming efficient and promising (Hussain et al., 2012). It has been linked with the development of microorganisms which may have integrated genetic information from higher plants, thereby ensuring better adaption to their host and they may perform some functions such as protection from insects, pathogens, and animals (Gouda et al., 2016).

Infectious and parasitic diseases are responsible for almost half of the death rate all over the world (Menpara and Chanda, 2013). Endophytes have been reported as the source of many bioactive compounds and several secondary metabolites available commercially today (Singh and Dubey, 2015). Endophytic microorganisms are a depot of new metabolites that can be used as antimicrobial, antiarthritic, anticancer, immunosuppressant, and anti-insect drugs (Jalgaonwala et al., 2011; Godstime et al., 2014). As at present, just a few plants have been studied for endophytes diversity and ability to produce bioactive secondary metabolites. Recent studies have reported that novel bioactive compounds produced by most endophytic microorganisms are important in overcoming the problem of antibiotic resistance by most pathogenic microorganisms (Godstime et al., 2014). Numerous bioactive compounds like vinblastine, amptothecin, hypericin, podophyllotoxin, camptothecin among others produced by endophytes have already been commercialized and have been found useful in agriculture and pharmacology (Joseph and Priya, 2011; Zhao et al., 2011).

## Anticancer Activity and Compounds

Cancer is a disease identified by the uncontrolled multiplication of abnormal cells which results in death in human beings when not controlled. Globally cancer prevalence is said to have increased to 9.6 million deaths and 18.1 million cases in the year 2018 (Toghueo, 2019). All over the world, those who survive cancer disease within 5 years of its detection are approximated as 43.8 million (Toghueo et al., 2019). In 2004 cancer was said to be responsible for about 13% (estimated to be 7.4 million) of the world death (Gouda et al., 2016). The drugs used in the treatment of cancer show non-specific toxicity for the multiplying normal cells have many side effects and many are still not active in the treatment of some cancer forms (Pasut and Veronese, 2009). The discovery of metabolites with cytotoxic properties has given insights in anticancer therapy for some decades (Pimentel et al., 2011). Endophytes have been reported to have the ability to produce novel metabolites that can serve as anticancer agents (Rajamanikyam et al., 2017). A summary of related studies on the anticancer properties of endophytes is presented in Table 2.

**Table 2:** Summary of studies on the anticancer prospects of endophytes.

**Endophytes**	**Compound secreted**	**Class of compound**	**Activity**	**Cell active against**	**References**
**Endophytic fungi**					
*Fusarium oxysporium*	Vincristine	Alkaloids	Anticancer	–	Zhang et al., 2000
*Mycellia sterilia*	Vincristine	Alkaloids	Anticancer		Yang et al., 2004
*Enthrophospora infrequens*	Camptothecin	Quinolone alkaloid	Anticancer		Puri et al., 2005
*Phomopsis cassiae*	3,12-dihydroxydalene 3,12-dihydroxycalamenene 3,11,12-trihydroxycadalene	Terpenoids	Anti-proliferative	HeLa cervical cells	Silva et al., 2006
*Periconia atropurpurea*	EtOAc extract	–	Cytotoxicity	–	Teles et al., 2006
*Garcinia* sp.	EtOAc extract	–	Antiproliferative and cytotoxicity	Vero cell lines	Phongpaichit et al., 2007
*Collentotrichum gloesporiodes*	Taxol	Alkaloids	Cytotoxicity	Human cancer cells lines BT220, int 407, H116, HLK 210, HL251.	Gangadevi and Muthumary, 2008
*Aspergillus fumigatus*	Cytotoxic alkaloids	Alkaloids	Cytotoxicity	Leukemia cancer cell line	Konecny et al., 2009
*C. gloesporiodes*	Taxol	Alkaloids	Anticancer		Nithya and Muthumary, 2009
*Alternaria alternata*	EtOAc extract	–	Antitumor and cytotoxicity	HeLa cells	Fernandes et al., 2009
*Alternaria* sp.	Xanalteric acids	Phenols	Cytotoxicity		Kjer et al., 2009
*Fusarium solani*	Camptothecin	Quinolone alkaloid	Anticancer	–	Shweta et al., 2010
*Lasidiplodia theobromae*	Taxol	Alkaloids	Anticancer	MCF-7	Pandi et al., 2011
*Cephalotheca faveolata*	Sclerotiorin	Polyketides	Anticancer	Colon cancer (HCT-116)	Giridharan et al., 2012
*Phoma* sp.	5-hydroxyramulosin	Polyketides	Anticancer		Santiago et al., 2012
*Penicillium* sp.	Arisugacin	Terpenoid derivatives	Anticancer	HeLa, HL-60, and K562 cell lines	Sun et al., 2014
*A. flavus*	Solamargine	Steriods	Cytotoxicity	–	El-Hawary et al., 2016
*Taxomyces andreanae*	Paclitaxel	Alkaloids	Anticancer	–	Alurappa et al., 2018
*Chaetomium* sp., *Alternaria* sp., and *Collentotrichum* sp.	EtOAc extract	–	Cytotoxicity	HeLa and MCF-7 cells	Dhayanithy et al., 2019
**Endophytic actinomycetes**					
Streptomyces thermoviolaceus TP-A0648	Anicemycin	Alkaloids	Antitumor	–	Igarashi, 2004
*Streptomyces* sp*. SUC1*	Lansai A-D	Alkaloids	Anticancer	–	Tuntiwachwuttikul et al., 2008
*Actinosynnema pretiosum*	Ansamitocin	Polyketides	Antitumor	–	Siyu-Mao, 2013
*Micromonospora lupini Lupac 08*	Lupinacidin C	Quinones	Antitumor	Murine colon carcinoma cells	Igarashi et al., 2011
*Streptomyces* sp*. CS*	Naphtomycin A	Quinones	Antitumor	P388 and A-549 cell lines	Lu and Shen, 2007
*Streptomyces laceyi MS53*	6-alkalysalicilic acids, salaceyins A and B	Fatty acid derivatives	Anticancer	–	Singh and Dubey, 2018
**Endophytic bacteria**					
*Acinetobacter guillouiae*	EtOAc extract	–	Anticancer	U87MG glioblastoma and A549 lung carcinoma cells	Sebola et al., 2019
*Bacillus subtilis PXJ-5, Bacillus* sp*. CPC3, Bacillus cereus strain ChST*	Camptothecine	Alkaloids	Anticancer	–	Shweta et al., 2013
–	EtOAc extract	–	Cytotoxic	A549 lung cancer cell lines	Swarnalatha and Saha, 2016
*Pantoea* sp.	EtOAc extract	–	Anticancer	A549 lung carcinoma and UMG87 glioblastoma cell lines	Uche-Okereafor et al., 2019

## Antioxidant Activity and Compounds

The major significance of antioxidant compounds is the fact that they are very active in controlling diseases linked to the presence of oxygen-derived free radicals and ROS, which may be responsible for the degeneration of cells, DNA damages, and carcinogenesis (Mishra et al., 2014). Antioxidants are now considered as promising alternatives in the treatment and prevention of diseases linked with ROS such as Diabetes mellitus, cancer, hypertension, Alzheimer's disease, Parkinson's disease, ischemia, and atherosclerosis. Most antioxidants have antiatherosclerotic, anti-carcinogenic, anti-inflammatory, antitumor, and antimutagenic activities both in small and large quantities (Hood and Shew, 1996; Mishra et al., 2014).

A phenolic metabolite identified as Graphislactone A, produced by *Cephalosporium* species, also, IFB-E001 found inside *Trachelospermum jasminoides* was found to have the ability to search for free radical and it exhibited stronger antioxidant properties than ascorbic acid and butylated hydroxytoluene (BHT) coassayed in the research (Suryanarayanan et al., 2009). Shoeb et al. (2014) also reported that an endophytic fungus Penicillium thiomii produced an antioxidant identified as terminatone. The crude extracts of *Rhodiola* spp. were also reported to scavenge DPPH, ${\text{O}}_{2}^{-}$, and OH radicals, and also in chelating Fe^2+^ (Cui et al., 2015). EtOAc extract of *Diaporthe* spp. was found to produce a strong antioxidant (Toghueo, 2019).

A novel compound called sesquiterpene isolated from *Acremonium* sp. also showed strong antioxidant activity (Elfita et al., 2012). Ethyl acetate extract of endophytic fungus *Fennellia nivea* had a notable quantity of total phenolics which might be responsible for its high antioxidant activity (Saraswaty et al., 2013). *Aspergillus fumigatus* SG-17 was found to secrete a compound called (Z)-N-(4-hydroxystyryl) formamide (NFA), an equivalent of coumarin which functions as an antioxidant both *in vitro* and *in vivo*. Subsequent analysis through MS and NMR further established the claim (Qin et al., 2019). A summary of related studies on the antioxidant properties of endophytes is presented in Table 3.

**Table 3:** Summary of studies of antioxidant properties of endophytes.

**Endophytes**	**Host plant**	**Compound secreted**	**Class of compounds**	**References**
**Endophytic fungi**				
Strain AcapF3	*Tabernaemontana divaricata* (L), *Rauvolfia verticillata* (Lour.)	–	Phenol	Huang et al., 2007
*Aspergillus* sp.	*Calotropis procera, Catharanthus roseus, Euphorbia prostrate, Vernonia amygdalina*, and *Trigonella foenum-graecum*	Gallic acid	Phenol	Khiralla et al., 2015
*Aspergillus minisclerotigens* AKF1 and *Aspergillus oryzae* DK7	*Mangifera casturi* Kosterm	Dihydropyran and 4H-Pyran-4-one, 5-hydroxy-2-(hydroxymethyl-(CAS) Kojic acid	–	Nuraini et al., 2019
*Rhodiola* spp.	Alpine plants	Salidrosides, p-tyrosol, and rosavins	Phenolic and flavonoid	Cui et al., 2015
*Phoma* sp., *Colletotrichum spiralis, Chaetomium* sp.	–	MeOH extract	Phenol	Singla, 2019
Penicillium citrinum CGJ-C1, *P. citrinum* CGJ-C2, *Cladosporium* sp. CGJ-D1, and *Cryptendoxyla hypophloia* CGJ-D2	*Tragia involucrata* Linn	L-ascorbic acid	–	Danagoudar et al., 2018
*Aspergillus niger, A. flavus, Alternaria alternata*	*Lannea coromendalica*	EtOAc extract	Phenolic compound	Premjanu and Jaynthy, 2014
*Chaetomium globosum*	*Adiantum capillus*	EtOAc extract	Phenolic compound	Selim et al., 2018
*Phyllosticta* sp.	*Guazuma tomentosa*	EtOH extract	Phenol	Srinivasan et al., 2010
**Endophytic bacteria**				
*Methylobacterium radiotolerans* MAMP 4754	*Combretum erythrophyllum*	EtOAc extract, Chloroform extract	Alkaloids, flavonoids, Phenol and steroids	Photolo et al., 2020
*Lactobacillus* sp.	*Adhathoda beddomei*	EtOAc extract	Phenolic compounds	Swarnalatha et al., 2015
*Pseudomonas hibiscicola, Macrococcus caseolyticus, Enterobacter ludwigii, Bacillus anthracis*	*Aloe vera*	EtOAc extract	Alkaloids and flavonoids	Akinsanya et al., 2015
*Pseudocercospora* sp. ESL 02	*Elaeocarpus sylvestris*	Terreic acid (1) and 6-methylsalicylic acid	–	Prihantini and Tachibana, 2017
EC3	*Carica papaya* L.	Gallic acid	Phenolic compounds	Sarjono et al., 2019
**Endophytic actinomycetes**				
*Streptomyces aureofaciens* CMUAc130	*Zingiber officinale*	5,7-Dimethoxy-4-pmethoxylphenylcoumarin; 5,7-Dimethoxy-4-phenylcoumarin	Coumarins (Alpha Benzopyrones)	Taechowisan et al., 2007
*Streptomyces* sp. Tc052	*Alpinia galanga*	Kaempferol, Isoscutellarin, Umbelliferone, and Cichoriin	Flavoniods	Singh and Dubey, 2018
*Micromonospora* sp. PC1052	*Puereria candollei*	S-adenosyl-Nacetylhomocysteine	Peptides	Boonsnongcheep et al., 2017
*Streptomyces* sp. MS1/7	–	2-Allyloxyphenol	Phenol	Singh and Dubey, 2015

## Antidiabetic Activity

Nature has given us many natural resources which can be used for medicinal purposes. The hypolipidemic and antidiabetic prospects of endophytic fungi extracts from *Salvadora oleoides in* Wistar albino rats induced with diabetes when loaded with glucose and alloxan was examined (Dhankhar et al., 2013). Glucose tolerance test showed that extracts from endophytic fungi such as *Phoma* sp. and *Aspergillus* sp. successfully reduced the glucose level in the blood of the rats. Akshatha et al. (2014) assessed antidiabetic prospects of endophyte extracts from the tissue of *Rauwolfia densiflora* and *Leucas ciliate*, two of the most prominent medicinal plants used in treating diabetes. The result showed that α-amylase inhibitor slows down the glucose from dietary complex carbohydrate and retards the rate at which glucose is absorbed. Also, Kaur (2018) screened endophytic fungi for their ability to act as for alpha-glucosidase inhibitors. It was reported for the first time that extracts from *Fusarium* sp. and *Alternaria* sp. act as alpha-glucosidase inhibitors, the study establishes endophytic fungi as sources of pharmaceutically important molecules.

*Xylariaceae* sp. also secreted a coumarone compound purified as 8-hydroxy-6,7-dimethoxy-3-methylisocoumarine which was reported to have been active against α-glycosidase (Indrianingsih and Tachibana, 2017). Pujiyanto et al. (2012) revealed that the crude extracts of an endophytic bacterium identified as *Streptomyces olivochromogenes* which showed potential antidiabetic activity. Three compounds (*S*)-(+)-2-*cis*-4-*trans*-abscisic acid, 7′-hydroxy-abscisic acid and 4-des-hydroxyl altersolanol A secreted by *Nigrospora oryzae* reported to be active against α-glycosidase (Uzor et al., 2017). GancidinW (GW) secreted by *Streptomyces paradoxus* VITALK03 was also reported to be active against α-glycosidase (Ravi et al., 2017).

## Immunosuppressive Activity

There have been ongoing studies on how to identify an effective agent for the suppression of immunological disorders especially autoimmune diseases and graft rejection (Rajamanikyam et al., 2017). *Fusarium subglutinans* an endophytic fungus was found to secrete subglutinol A and B which act as an immunosuppressive agent. The drug produced from it is used to avert the problem of allograft rejection in patients who undergo a transplant and it is promising in the treatment of autoimmune diseases like insulin-dependent diabetes and rheumatoid arthritis (Padhi et al., 2013). An antifungal peptide compound called Pseudomycins which was reported to be active against human pathogen *Candida albicans* found to contain special amino acids like L-chlorothreonine, L-diaminobutyric acid, and L-hydroxyl aspartic acid (Castillo et al., 2003).

Ambuic acid which is a cyclohexenone belongs to the family of pseudomycins which was secreted by *Pestalotiopsis microspore* and found to be active against human pathogens. A bioactive agent from *Streptomyces* species identified as ambuic acid was effective against both gram-negative and gram-positive bacteria (Suryanarayanan et al., 2003). Crude extracts of fungi endophyte, *Penicillium* sp. ZJ-SY_2_, showed strong immunosuppressive activity when structural elucidation was done using 1D, MS, 2D, and NMR data. Compounds 1, 3, 5, and 7 showed strong immunosuppressive activity using IC_50_ values ranging from 5.9 to 9.3 μg/mL (Liu et al., 2016). Three novel derivatives of xanthone, including two earlier reported to contain sulfur as natural products: sydoxanthone A (1) and sydoxanthone B (2), and 13-O-acetylsydowinin B (3) were found to be secreted by an endophytic fungus, *Aspergillus sydowii*. Structural elucidation was done by, UV, MS and NMR data to establish the data. *In vitro* suppression assay carried out on LPS-induced and Con A proliferation of splenic lymphocytes of a mouse showed that the compounds have moderate immunosuppressive activities (Song et al., 2013).

Chloroform (CEEI) and methanolic extracts produced by *Entrophospora infrequens* exhibit delayed-type hypersensitivity (DTH) reactions (Pur et al., 2007). Three compounds isolated from *Pestalotiopsis leucothës* were found to be effective on T and B-cells and monocytes (Kumar et al., 2005). Madagundi et al. (2013) isolated endophytic fungi from *Ocimum sanctum* Linn and assessed their extracts *in vitro* for immunomodulatory properties on human polymorphonuclear (PMN) cells such as phagocytosis. The immunosuppressive assay of Curtachalasin secreted by an endophytic fungus *Xylaria* cf. curta against cell proliferation of concanavalin A (ConA) induced T lymphocyte cell and lipopolysaccharide (LPS) induced B lymphocyte cell proliferation was reported by Wang et al. (2019). The crude extracts of *Brevibacterium* sp. YXT131 an endophytic actinobacterium modulated the immune response by reducing the proinflammatory cytokines interleukin (IL)-12/IL-23 p40 in the serum of mice (Wei et al., 2018). The use of bioagents in immune modulation of some diseases is a current and novel research area.

## Antiviral Activity

The discovery of promising antiviral compounds for endophytes is still novel. There are still limited numbers of compounds that have been attributed to endophytes. The limiting factor in the production of antiviral compounds by endophytes is the fact that no antiviral screening systems exist. Most antibiotics products from endophytic fungi are known to strongly inhibit viral growth. The elucidation using mass spectrometry and NMR methods showed that two cytomegalovirus protease inhibitors in human and cytonic acids A and B were effective against the growth of viruses (Harper et al., 2001). Some metabolites secreted by endophytes from desert plants serve as a promising source in identifying potent inhibitors in the replication of HIV-1 (Wellensiek et al., 2013).

*Alternaria tenuissima* QUE1Se was reported to produce an antiviral compound called Altertoxins which was found to be effective against HIV-1 virus (Bashyal et al., 2014). Also, many extracts from endophytic fungi were tested against the replication of HIV-1 virus in T-lymphocytes, four extracts were found not to be toxic but inhibited the virus with differences ranging from 75 to 99%. Three of the extracts were fractionated and the DB-2 fraction was observed to completely inhibit the replication of HIV-1 (Wellensiek et al., 2013). Compounds extracted from *Emericella* sp. (HK-ZJ), namely aspernidine (A, B), dehydroaustin, emeriphenolicins (A, D), austinol, emerimidine (A, B), austin, and acetoxy dehydroaustin were reported to confer antiviral activity against the influenza A virus (H*1*N*1*) (Zhang et al., 2009). Extracts from endophytic fungi species Aspergillus, *Pestalotiopsis, Fusicoccum, Phomopsis, Guignardia, Penicillium*, and *Muscodor* were also assessed for their antiviral activity against Herpes simplex virus type 1 (HSV-1 ATCC VR-260), many of the fungi species showed weak to moderate antiviral activity (Phongpaichit et al., 2007). Also, crude extracts from 81 endophytic fungi isolated from many medicinal plants showed antiviral activity (Rajamanikyam et al., 2017). Recently, extracts from some endophytic actinomycetes were reported to possess antiviral properties, for example, metabolites from *Streptomyces* sp. GT2002/1503 exhibited antiviral activity against R5 tropic HIV infection (Ding et al., 2010). *Jishengella endophytica* 161111 also secreted an antiviral compound, 2-(furan-2-yl)-6-(2S,3S,4- trihydroxybutyl) pyrazine which was active against influenza A virus subtype H1N1 (Wang et al., 2014).

## Antiarthritis and Anti-Inflammatory Activities

The immune system of an individual plays an active role in certain deadly diseases, a hyperactive immune system may result in diseases such as arthritis. Rheumatoid arthritis (RA) is an inflammatory and autoimmune disease that is systemic with symptoms such as swelling, pain, bone, and cartilage destruction which can later lead to permanent disability. Surprisingly, the exact causative agent of the disease is not known. Most researchers are currently looking for more medicinal agents from microbes because the present synthetic drugs are very costly and have many side effects (Choudhary et al., 2015). An endophytic fungus, *Talaromyces wortmannii*, isolated from *Aloe vera* secreted some bioactive metabolites which showed active anti-inflammatory activity. This ability is because a metabolite produced by the organism inhibit the release of IL-8 by blocking the activation of AP-1 and NF-_k_B (Pretsch et al., 2014). Methanolic extracts for *Lepidosphaeria* sp., an endophytic fungus, also showed anti-inflammatory activity and it is promising as a drug which might be adopted for the treatment of inflammatory diseases like rheumatoid arthritis (Shah et al., 2015). The main reason for screening endophytes is to establish new inhibitors for pro-inflammatory cytokines which are encountered in many immunological pathways. Extracts of endophytic fungi isolated from *Mimusops elengi* (bakul), an important medicinal plant in India also showed strong anti-inflammatory activity (Deshmukh et al., 2009). Methanolic extract of *Chaetomium globosum* was observed to be responsible for improved arthritis and mobility scores, and was concluded to possess a strong inhibitory effect on the morphological features of rheumatoid arthritis in an adjuvant-induced rat model (Abdel-Azeem et al., 2016).

## Antimalarial Activity

Malaria is still one of the major causes of mortality and morbidity in the world with over 3.3 billion people living with the ongoing risk of transmission (Ateba et al., 2018). In 2016, about 91 countries reported ~216 million new cases of malaria and 445,000 mortalities. The people most affected by malaria are those people that live in the subtropical and tropical regions of the world, people from Southeast Asia and sub-Saharan Africa where ~80% of cases of malaria recorded are traceable to *Plasmodium falciparum* (Ateba et al., 2018). The recent widespread of anti-drug resistant malaria parasites makes the search for alternative and new malarial treatment drugs urgent (D'alessandro, 2009).

Munumbicins E-4 and E-5 produced by endophytic fungi showed antimalarial activity, which was found to have double the effect of chloroquine (Suryanarayanan et al., 2003). An endophytes *Diaporthe miriciae* was found to produce a secondary metabolite called epoxycytochalasin H which expresses strong antimalarial inhibition against a strain of *Plasmodium falciparum* that is resistant to chloroquine (Ferreira et al., 2017). A report by Ateba et al. (2018) showed that endophyte species *Paecilomyces lilcinus* and *Penicillium Janthinellum* are storehouse of novel metabolites that are active against *Plasmodium falciparum* and promising in the cure of malaria. Endophytic fungi, *Fusarium* sp. and *Nigrospora* sp. were also reported to secrete bioactive metabolites which showed antiplasmodial activity against *Plasmodium falciparum* (Kaushik et al., 2014).

## Antituberculosis Activity

Tuberculosis (TB) is a globally recognized communicable disease with the etiological agent as *Mycobacterium tuberculosis* which often affects the lungs. It has been one of the major disease troubling human beings for centuries. Death rate as a result of TB infection is estimated at two billion globally with almost nine million new cases emerging every year (Tsara et al., 2009). Tuberculosis is responsible for more deaths of otherwise healthy people than diseases that are infectious such as malaria and AIDS (Corbett et al., 2003). The challenge with TB is that there is no effective treatment method for the disease. However, with the advent of Multi-drug resistance strains of *M. tuberculosis*, the disease has established itself as a major source of concern to humans (Khunjamayum et al., 2017).

**Figure 3:**
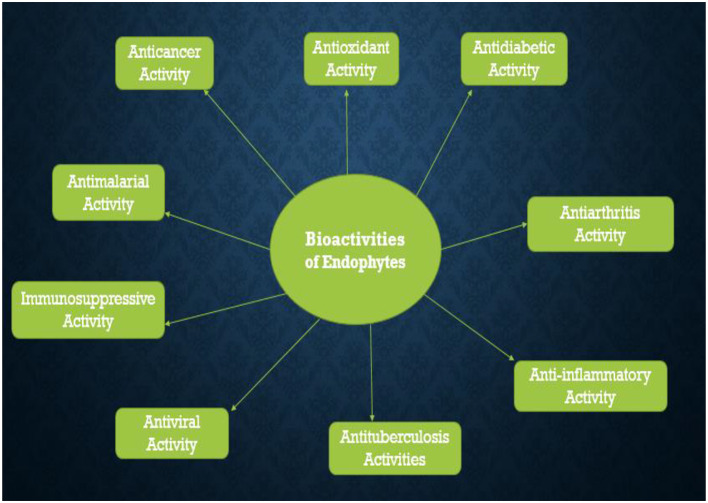
Biological activities of importance to humans present in endophyte's metabolites.

Endophytes are capable of secreting some bioactive compounds that can successfully inhibit the prevalence of TB caused by *M. tuberculosis*. Endophytic fungi species, *F. solani* and *C. gleosporoides* isolated from *G. glabra* showed strong inhibition against *Mycobacterium tuberculosis* strain H37Rv with MIC of 18.5 and 75 μg/ml, respectively (Shah et al., 2016). The crude extracts of endophytic bacteria, *Streptomyces* sp. and *Bulkholderia fungorum* were reported to show great inhibition against the pathogenic strain of *Mycobacterium tuberculosis* and the IC50 values recorded for them were <100 μg/ml (Khunjamayum et al., 2017). The diverse biological activities of endophytes are presented in Figure 3 below.

## Future Prospects

Concise studies on a specific population of endophytes active in a host are required before bulk production can be carried out which often requires research with advanced technology. Studies are also needed as regards getting plant-specific inoculum doses of endophytes, this will help in reducing bulk production and also enhance productivity by reducing our dependence on synthetic fungicides, pesticides, and fertilizers. Development of endophytes that can be sprayed just like most chemical pesticides will help in the acceptance of endophytes in integrated pest management.

Future studies will need to take into account the development of genomic tools and metabolomics tools for further studies on how endophytes colonize the plant and plant-microbe interaction. There is still a need to study the compounds produced by endophytes and their activities in reducing diseases. This will help in developing efficient markers for some important and distinct biocontrol agents and assessing the effects of plant genotypes, innate microbe community, and most importantly the environment. This structured approach will also help in discovering new endophytes with important traits.

Molecular study of these endophytes is important in order to improve drug research. Also, metagenomics study will be very important in order to showcase the diversity of endophytes and the functions they are capable of performing through a detailed analysis of their genes. Molecular biology techniques can be used for the isolation and identification of the different types of genes present in the biosynthetic pathways and this will further open our eyes to new bioactive compounds at a commercial level as well as in the laboratory. Future studies should focus on the biosynthetic pathways which might be responsible for the secretion of numerous important bioactive compounds by endophytes.

Also, future studies can look into the development of endophytic nanoparticles which will help in improving the plant growth. Transfer of genes can also be employed in order to detect more efficient species. The idea of manipulating genes can help the host plants in developing new traits like phytoremediation and herbicide resistance, among others, which could more suitably regulate metabolism. There is no microbial technology that can be considered successful until it has been commercialized. The specificity of endophytes within a plant is one of the limitations in its large scale production.

## Conclusion

The study attempts to appreciate the diverse mechanisms used by endophytes in protecting plants from diseases for sustainable agriculture. Endophytic microbes support the plant and accelerate plant growth by employing different mechanisms of action, both direct and indirect. The major benefit of embracing such beneficial microorganisms in the field of agriculture is to bring about reduction in the use of different agrochemicals such as pesticides, chemical fertilizers, other artificial chemicals etc. and this would make agriculture more productive and sustainable. Endophytes can still be very useful in the biomedical field because endophytes can synthesize and secrete chemicals which may be used for the development of antibiotics of importance for human use. Many studies are still ongoing toward assessing the ability of endophytes to secrete novel bioactive compounds which will be of great importance in the treatment of human diseases.

Besides the numerous applications of endophytes in medicine, therapeutics, and mining, some novel metabolites may be useful in sustainable agriculture and in enhancing plant growth. These metabolites can also confer insecticidal, and pest control activities, alongside enhance plant nutrient uptake under extreme conditions such as drought, salinity, and waterlogging. Taken as a whole, novel bioactive compounds secreted by endophytes especially endophytic actinomycetes could offer immense contributions in address the present and future challenges in agriculture, environment and medicine. Finally, the application of metagenomics combined with next-generation sequencing technologies is expected to open up the numerous unexplored pool of antimicrobials secreted by yet uncultivated endophytic microbes.

## Author Contributions

AF collected the data and developed the first draft. OB conceived the research topic from which the review emanates and guided in the data acquisition and critically reviewed the various versions of the work. Both authors have carefully read the final manuscript and have agreed to the publication.

## Conflict of Interest

The authors declare that the research was conducted in the absence of any commercial or financial relationships that could be construed as a potential conflict of interest.
